# Influence of Scanning Strategy on Residual Stresses in Laser-Based Powder Bed Fusion Manufactured Alloy 718: Modeling and Experiments

**DOI:** 10.3390/ma17246265

**Published:** 2024-12-21

**Authors:** Carl-Johan Hassila, Andreas Malmelöv, Carl Andersson, Johan Hektor, Martin Fisk, Andreas Lundbäck, Urban Wiklund

**Affiliations:** 1 Applied Materials Science, Uppsala University, SE-751 03 Uppsala, Sweden; 2 Department of Engineering Sciences and Mathematics, Luleå University of Technology, SE-971 87 Luleå, Swedencarl.andersson@ltu.se (C.A.); 3 Department of Materials Science and Applied Mathematics, Malmö University, SE-205 06 Malmö, Sweden; johan.hektor@mau.se (J.H.); martin.fisk@mau.se (M.F.); 4 Division of Solid Mechanics, Lund University, P.O. Box 118, SE-221 00 Lund, Sweden

**Keywords:** PBF-LB, LPBF, EBSD, synchrotron diffraction, finite element method, mechanism-based material model, inherent strain, validation

## Abstract

In additive manufacturing, the presence of residual stresses in produced parts is a well-recognized phenomenon. These residual stresses not only elevate the risk of crack formation but also impose limitations on in-service performance. Moreover, it can distort printed parts if released, or in the worst case even cause a build to fail due to collision with the powder scraper. This study introduces a thermo-mechanical finite element model designed to predict the impact of various scanning strategies in order to mitigate the aforementioned unwanted outcomes. The investigation focuses on the deformation and residual stresses of two geometries manufactured by laser-based powder bed fusion (PBF-LB). To account for relaxation effects during the process, a mechanism-based material model has been implemented and used. Additionally, a purely mechanical model, based on the inherent strain method, has been calibrated to account for different scanning strategies. To assess the predicted residual stresses, high-energy synchrotron measurements have been used to obtain values for comparison. The predictions of the models are evaluated, and their accuracy is discussed in terms of the physical aspects of the PBF-LB process. Both the thermo-mechanical models and the inherent strain method capture the trend of experimentally measured residual stress fields. While deformations are also adequately captured, there is an overall underprediction of their magnitude. This work contributes to advancing our understanding of the thermo-mechanical behavior in PBF-LB and provides valuable insights for optimizing scanning strategies in additive manufacturing processes.

## 1. Introduction

The laser-based powder bed fusion (PBF-LB) manufacturing process has attracted a lot of interest in recent decades, with a dramatic increase in publications in the field. One branch of research is the modeling of residual stresses in components. Two main modeling strategies on the macro scale that are based on the finite element method exist. The first strategy is a coupled thermo-mechanical modeling approach where the lumping of hatches and layers is commonly applied, and the second strategy is the inherent strain method (ISM).

The ISM was initially developed to predict residual stresses and distortions of welded components [1]. It offers a fast approach for predicting residual stresses and distortions. However, the ISM suffers from its sensitivity to mechanical boundary conditions and the geometry of the component [2]. Therefore, it was never successful as a predictive method in computational welding mechanics. The nature of AM is different from welding though. In PBF-LB, the process always starts with a base plate, and therefore, the external mechanical boundary conditions are more or less the same. The most common approach of the ISM when simulating PBF-LB is the assumption that each layer experiences the same inherent strain. This does not take the change in mechanical stiffness and thermal boundary conditions due to the change in geometry that the part is undergoing during the building process into consideration. Improvements in the method, both in terms of extraction and in terms of the application of inherent strains, have made ISM a viable choice to model residual stresses and distortions in components manufactured using PBF-LB [3,4,5,6,7,8].

Inherent strains can be obtained by calibration to macro-scale models by using experimental data as input to an optimization algorithm [5,6], or from a detailed thermo-mechanical model comprising only a few layers [7,8]. The resulting inherent strains can be applied using a layer-by-layer approach for macro-scale mechanical models [4,6,7,8]. The first approach gives an inherent strain that directly relates to the process parameters used in the PBF-LB process. The drawback is that the ISM must be re-calibrated if the process parameters are changed, which requires additional experimental work. The second approach introduces more complexity to the model. The advantage is that no further experimental work is needed to re-calibrate the inherent strains, instead, the detailed model can be re-used with different process parameters to obtain a new inherent strain.

Thermo-mechanical finite element models that examine the impact of scanning strategy on deformation and residual stresses in the PBF-LB manufacturing process for multi-layer models are largely absent from the literature. For instance, Parry et al. [9] investigated the effect of scanning strategy on residual stress in a single-layer simulation, while Cheng et al. [10] and Zhang et al. [11] each studied models with three layers.

In this work, the impact of different scanning strategies on the deformation and residual stresses in FE models that contain hundreds of physical layers are modeled using a thermo-mechanical model. This is made possible by utilizing different lumping approaches of the hatches and layers. The thermo-mechanical model utilizes a material model that takes stress relaxation mechanisms into account when calculating the material flow stresses. The thermo-mechanical model has been used in previous work by Malmelöv et al. [12] where the model parameters are calibrated. However, in that work, the lumping of entire layers is utilized, which renders the model incapable of resolving the impact of different scanning strategies. In addition to the thermo-mechanical model, an inherent strain model is employed for comparison. The results from the models regarding residual stresses are compared with high-energy synchrotron measurements. The predicted deformations from the computational models due to the release of residual stresses after partially cutting the part from the build plate are also compared with the measurements.

## 2. Experimental Methods

For manufacturing the samples in this work, an EOS M100 PBF-LB system from EOS GmbH, Krailing, Germany was used. The system uses a focused 45 μm 200 W Yb-fiber laser to achieve complete melting of the metal powders. A protective argon gas atmosphere was present during the entire PBF-LB process. To build the components, Sandvik’s Osprey^®^pre-alloyed 718 metal powders were used with the composition shown in Table 1, holding a powder size distribution specified to 20–53 μm. The powder was produced using vacuum induction melting followed by gas atomization using argon gas (VIGA).

**Figure 1:**
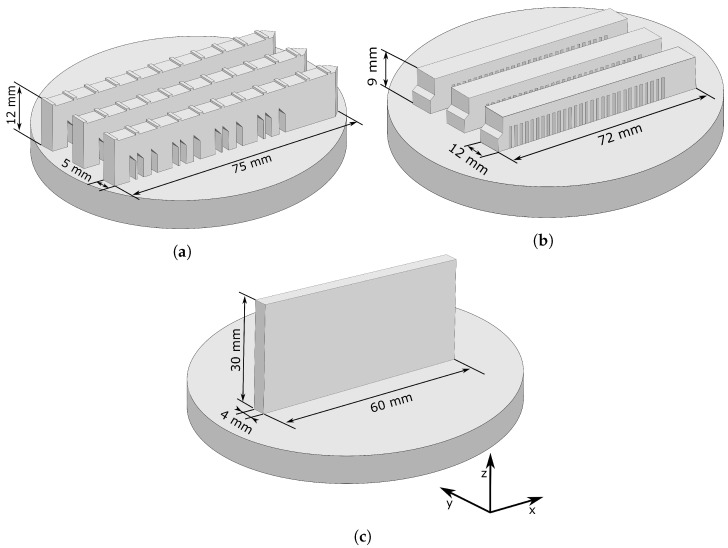
The printed geometries in this work: (**a**) the bridge, (**b**) the cantilever, and (**c**) the wall. The bridges and the cantilevers were printed as a set of three using either the scanning strategies XX or YY. The wall was printed using the YY scanning strategy.

Three sample geometries were printed applying a laser power of 160W, a scanning speed of 1400mm/s, and a hatch spacing of 60 μm. See Figure 1 for the geometries. The solidified layer was set to a nominal thickness of 20 μm, which resulted in an energy density of $95\;J/{\mathrm{mm}}^{3}$. This set of process parameters resulted in a material with a relative density greater than 99.9%, which was determined with optical microscopy on polished cross-sections.

Two scanning strategies were utilized for printing the components: XX and YY. The strategies are named after the scanning vectors and their direction with respect to the coordinate system. The XX scanning strategy has all individual hatch lines parallel to the x-axis, and the YY scanning strategy has all hatch lines parallel to the y-axis.

The geometry shown in Figure 1a, hereafter referred to as the ’bridge’, was printed in groups of three on two build plates using the bi-directional scanning strategies XX and YY. For the calibration of the inherent strain model, a cantilever geometry, later referred to as the ’cantilever’, was adopted from the Simufact Additive software suite (Hexagon, Stockholm, Sweden); see Figure 1b. The cantilevers were printed in groups of three on two build plates using the scanning strategies XX and YY. For evaluation of the residual stresses within the printed material, a geometry, hereafter called the ’wall’, was printed. The geometry of the wall is shown in Figure 1c. The wall was printed using the YY scanning. The YY strategy was chosen because it results in the shortest hatch length and ensures a stable printing process. A triple contour was used for the wall to achieve a better surface finish.

Microstructural investigation of the samples produced includes inverse pole figures (IPFs) and grain boundary maps obtained using EBSD. These measurements were conducted on a scanning electron microscope (SEM) containing a Nordlys Max EBSD detector from Oxford Instruments NanoAnalysis, High Wycombe, UK. An acceleration voltage of 20kV was used with a beam current of 2nA. The samples were prepared from small cubes cut from the printed wall. Measurements were performed on the yz, xz, and xy-planes (see Figure 1 for coordinates) to visualize the different grain morphologies.

### 2.1. Deflection Measurements

**Figure 2:**
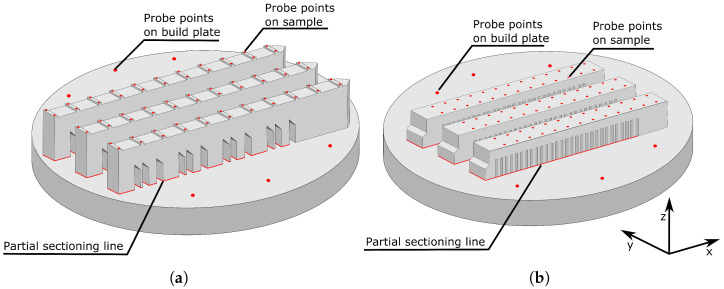
The deflection measurement points on the bridges (**a**), the cantilevers (**b**), and the build plates.

To ensure well-defined surfaces for subsequent measurements, the top surfaces of the printed geometries were flattened using a plane grinder after printing the geometries of the bridge and cantilever. Subsequently, each build plate was mounted in a fixture on the measurement table of a Coordinate Measurement Machine (CMM). This fixture allowed for the mounting and remounting of build plates with high accuracy. The measurement points are indicated in Figure 2 by red points, allowing the heights relative to the build plate to be calculated. After initial measurements, the printed geometries were partially sectioned from the build plate using an abrasive cutting wheel, allowing the printed geometries to deflect as a result of residual stresses. The build plates were then remounted for deflection measurements. 

### 2.2. Residual Stress Measurements

Synchrotron X-ray diffraction measurements were performed to spatially map the residual stress and strain fields in the printed walls. The experiment was conducted at the Swedish Materials Science beamline (P21.2) of the PETRA III synchrotron at DESY [13]. A $0.4\times 0.4\;{\mathrm{mm}}^{2}$ beam of 100 keV X-rays was raster scanned along the x- and z-directions (cf. Figure 1c) in steps of 2mm. The first point of the raster was located 1mm from the top of the wall and 1mm from its left edge. For each point in the raster, the wall was rotated relative to the incoming beam at angles $\psi_{0}$ ranging from $-45^{{}^{\circ}}$ to $+45^{{}^{\circ}}$ in increments of $5^{{}^{\circ}}$, with $0^{{}^{\circ}}$ indicating the position where the wall is normal to the incoming beam. The two-dimensional diffraction patterns were measured on a VAREX XRD 4343CT detector from Varex Industrial, Salt Lake City, UT, US placed 1.75m downstream of the sample. The geometry of the experiment was calibrated using a ${\mathrm{LaB}}_{6}$ reference sample.

**Figure 3:**
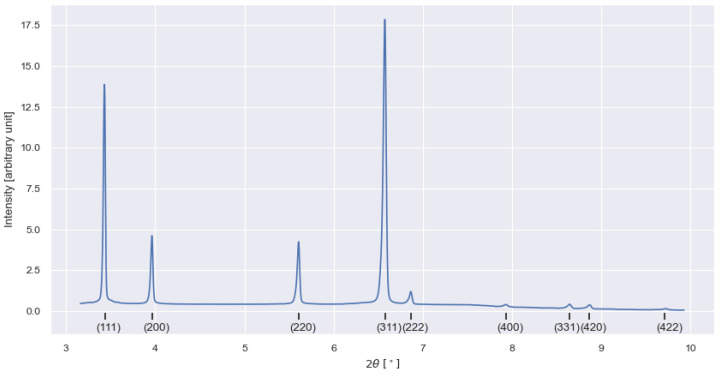
One-dimensional diffraction pattern from the wall with crystallographic planes identified.

The 2D diffraction patterns were caked in $5^{{}^{\circ}}$ bins using the Python package pyFAI 2023.03 [14]. The caking produces 72 1D patterns for each 2D image. An example of such a 1D pattern is shown in Figure 3. The Bragg angle (2θ) corresponding to the (311) reflection was identified in all 1D patterns by fitting a Pearson IV distribution to the data. The residual strain at each sample position is inferred by comparing the fitted Bragg angle to the Bragg angle of a stress-free reference sample. The reference sample had the shape of a comb (the same geometry as in [12]) and was taken from a wall built exactly the same way as the wall with residual stresses.

Assuming plane stress conditions, the elastic strain in each of the 72 azimuthal directions, α, can be expressed as [15]

(1) $$\varepsilon_{\alpha }=\frac{d_{\alpha }-d_{0}}{d_{0}}=\frac{\sin(\theta_{0})}{\sin(\theta_{\alpha })}-1=q_{1}^{2}\varepsilon_{xx}+q_{2}^{2}\varepsilon_{yy}+q_{3}^{2}\varepsilon_{zz}+q_{1}q_{3}\varepsilon_{xz}$$

where $q_{i}$ and $\varepsilon_{ij}$ are the components of the scattering vector [12] and the strain tensor, respectively, and *d* denotes the spacing of the lattice. The subscript 0 denotes quantities belonging to the strain-free reference sample. Evaluating Equation (1) for each of the 72 azimuthal directions for all the tilt angles $\psi_{0}$ results in an overdetermined system of equations containing 1368 equations that can be solved to extract the components of the strain tensor $\varepsilon_{ij}$. See Malmelöv et al. [12] for more details or Ramirez-Rico [16] for an overview of the applied full Debye ring-fitting method. The strain tensor is converted to stresses using Hooke’s law for plane stress.

The errors introduced in the measurement of stresses associated with single-tilt area-detector-based measurement techniques are discussed in [16]. The authors showed that determining the stresses with a relative error of 10% requires a standard deviation of $10^{-2}$ in a normally distributed error for 2θ. With the current setup, it is believed that this condition is satisfied. However, the errors resulting from misalignment have a significantly higher impact. These errors are more pronounced at low tilt angles ($\psi_{0}$), and decrease to be minimal or negligible values at tilt angle of $45^{{}^{\circ}}$. In the current setup, where the sample is rotated $\pm 45^{{}^{\circ}}$, any misalignment in the sample orientation tends to cancel out, thereby reducing the overall errors in the measurements.

## 3. Finite Element Model

**Figure 4:**
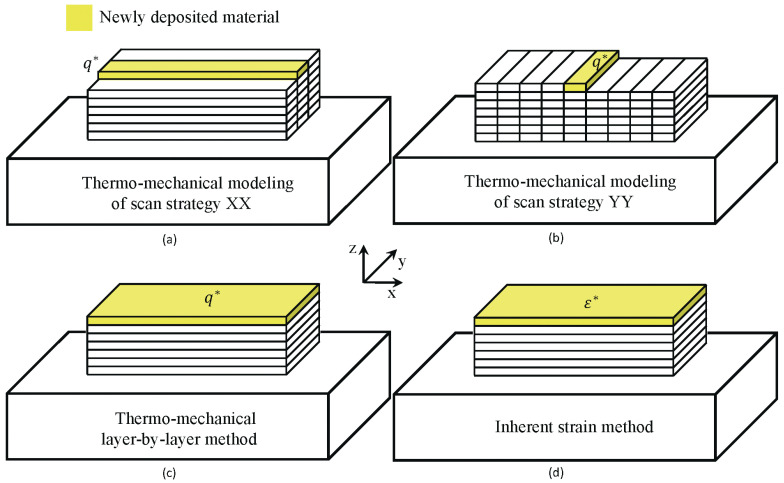
The different lumping techniques: (**a**) Hatch-by-hatch approach of scanning strategy XX. (**b**) Hatch-by-hatch approach of scanning strategy YY. (**c**) Layer-by-layer approach. The heat input, $q^{*}$, is calculated according to Equation (2). (**d**) The inherent strain approach with a purely mechanical model.

In the finite element analysis, two lumping techniques are employed. In the first approach, multiple physical layers and hatches are combined, as shown in Figure 4a,b. This approach, known as the ’hatch-by-hatch’ approach, aims to capture the influence of the scanning strategy on the modeling of stresses and deformations. In the second approach, several physical layers are grouped together, as depicted in Figure 4c. This approach, referred to as the ’layer-by-layer’ approach, cannot resolve the effects of the scanning strategy but offers a more computationally efficient alternative compared with the hatch-by-hatch approach. Finally, the deformation and residual stresses were simulated using the ISM (inherent strain method), which employs a lumping technique similar to the layer-by-layer approach, as shown in Figure 4d. This method represents the most computationally economical strategy among the modeling approaches.

**Figure 5:**
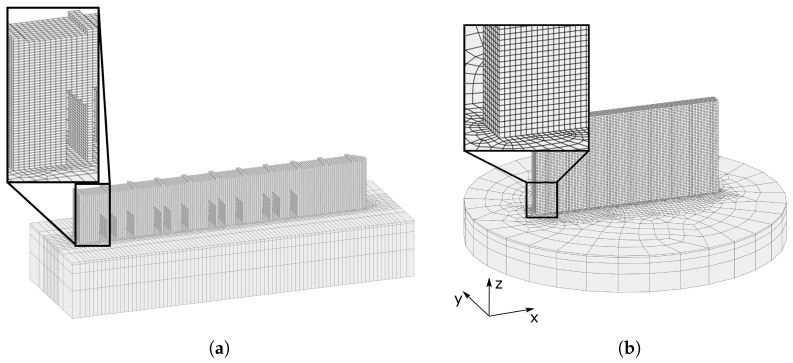
Mesh of (**a**) the bridge and (**b**) the wall for the thermo-mechanical model.

The finite element mesh for the bridge is shown in Figure 5a and contains 71,420 fully integrated eight-node hexahedron elements. In the physical printing, three identical bridges were built on the same build plate. To reduce the computation time, only one bridge is modeled on the build plate. The same mesh is used for both the hatch-by-hatch and layer-by-layer approaches. The bridges are modeled with 50 layers instead of the 625 physical layers. This corresponds to a modeled layer thickness of 0.25mm which in turn is the same thickness as the modeled layer in [12]. In the hatch-by-hatch approach, several hatches are lumped into 0.5mm wide sections. This results in that 10 and 149 modeled sections are added for each layer for scanning strategy XX and YY, respectively, compared with the physical number of hatches of 83 and 1250. See Table 2 for an overview of the number of printed and modeled layers and hatches.

The mesh of the wall is shown in Figure 5b and contains 127,804 fully integrated eight-node hexahedron elements. The wall is modeled with the inherent strain modeling approach, the thermo-mechanical layer-by-layer approach, and the hatch-by-hatch YY approach. The layer thickness of 0.5mm results in 60 modeled layers, which can be compared with the 1500 physical layers, while the hatches are lumped into 0.5mm wide sections. The modeled layer thickness is therefore twice as thick as for the bridge to decrease the simulation time.

### 3.1. Thermo-Mechanical Model

The thermo-mechanical simulations involve the utilization of the finite element software MSC.Marc 2021, employing a formulation designed to handle large strains and deformations. The coupling of thermal and mechanical analyses is achieved through a staggered approach [2]. The heat input model to the finite element model is approximated according to

(2) $$q^{*}=\frac{\eta Q}{t^{*}whv}$$

where η is the efficiency factor. It is related to the fraction of the laser power that is absorbed by the model. The parameter $t^{*}$ is a fictive heating time and is a result of the lumping approach. Other parameters in Equation (2) are related to the PBF-LB process parameters, where *Q*, *w*, *h*, and *v* are the laser power, the layer thickness, the hatch spacing, and the scanning speed, respectively. Applying the given heat input model in a finite element simulation will result in the same total energy as if all hatches and layers were modeled individually. After the addition of each lumped volume, the material is allowed to cool to near room temperature before adding the next hatch. This assumption is in accordance with [17,18]. Heat losses from the top surface are implicitly considered in the calculations by incorporating an efficiency factor. To model the heat loss to the surrounding powder, a heat transfer coefficient of $5\;W/m^{2}K$ is employed [12,19]. In the case of the build plate, where it comes into direct contact with the aluminum frame of the PBF-LB system, the heat transfer coefficient is significantly increased to $115\;W/m^{2}K$. The build plate in the EOS M100 system is kept in place by reducing the air pressure on the bottom side of the build plate. Due to the pressure difference on the top and bottom sides, the build plate is pressed down towards the frame of the machine. This ensures a firm positioning of the build plate with negligible mechanical impact. The mechanical boundary conditions are therefore applied to act as a simply supported plate. This allows for free thermal expansion, without inducing any additional thermal stresses, while rigid body motion is prevented.

**Figure 6:**
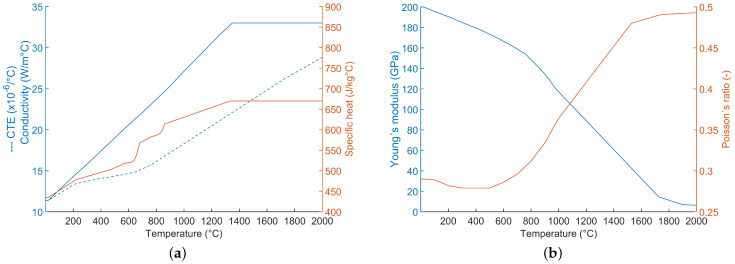
The temperature dependence of the material properties: (**a**) Coefficient of thermal expansion (CTE), thermal conductivity, and specific heat capacity coefficient. (**b**) Young’s modulus and Poisson’s ratio.

The transient heat transport equation that describes the temperature distribution in the workpiece [20] accounts for temperature-dependent material properties. The used thermal expansion coefficient (CTE) is based on data from [21], the conductivity is based on [22], and the specific heat is based on [23], and they are presented in Figure 6a, while Young’s modulus and Poisson’s ratio [22] are shown in Figure 6b. The data have been adjusted for higher temperatures. Lastly, it is assumed that the latent heat for melting and solidification is 240kJ/kg in the temperature interval 1160–1340 °C [24]. If the temperature reaches a higher value in the model than there is material data available for, then the cut-off method is applied [25].

The stress–strain response of the solidified material is described utilizing a mechanism-based, or physically based, plasticity model. It is based on the framework by Bergström [26] and Estrin and Mecking [27]. The main contribution to hardening is the dislocation density. The model has been adapted to several materials [28,29,30], with mechanisms relevant to each specific material. In the current work, the material model used is presented in Malmelöv et al. [31], which offers a comprehensive description of the model. Therefore, only a summary is given here.

The flow stress $\sigma_{y}$ is assumed to be the sum of hardening mechanisms such that

(3) $$\sigma_{y}=\sigma_{G}+\sigma_{HP}+\sigma^{*}+\sigma_{S}$$

where $\sigma_{G}$ is the effect of the interaction with the substructure of the immobile dislocation and is thus a function of the immobile dislocation density. $\sigma_{HP}$ is the grain size dependency (Hall–Petch), a hardening contribution caused by dislocation pile-ups at grain boundaries, and accounts for the energy needed for dislocations to cross grain boundaries. Consequently, smaller grains give a more rapid immobilization of the dislocations which results in flow stress hardening. In the model, a mean grain size of 20 μm is used. This value was determined in a previous work for a wall built with PBF-LB in alloy 625 [12]. The microstructure in that work is very similar to the microstructure in the current work and is believed to be a good representation here also. $\sigma^{*}$ is called the short-range contribution and describes the resistance to motion of dislocations in the presence of obstacles. As thermal vibrations can help dislocations pass such obstacles, the contribution of $\sigma^{*}$ is strongly temperature dependent. Lastly, $\sigma_{S}$ is an explicit contribution from solid solution strengthening.

**Figure 7:**
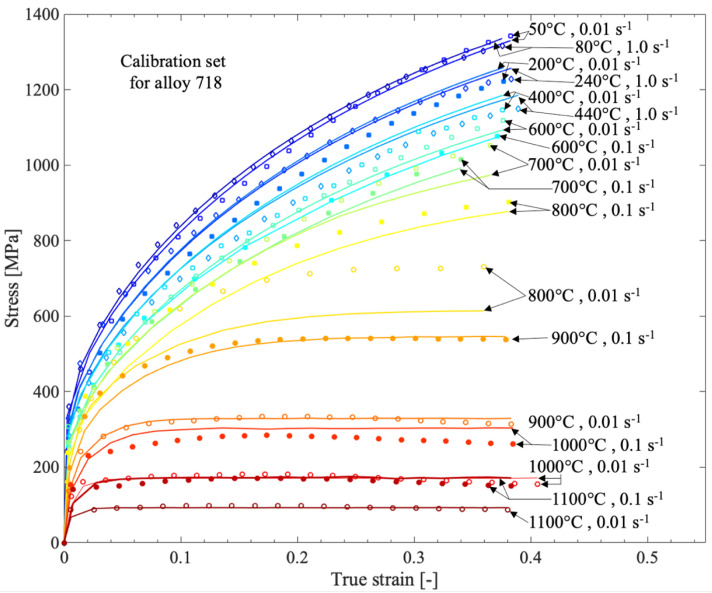
Calibration set for the mechanism-based material model. The figure is acquired from [31] and republished with permission from the authors.

The model for the yield stress has been implemented in a stress–strain algorithm in the finite element code, where a von Mises yield criterion is applied. For this, the yield stress and the hardening modulus must be given for the current plastic strain. The evolution model of the yield limit is based on the immobile dislocation density and is stored as an internal state variable for each time increment. With this method, the evolution of dislocation density is modeled in a way similar to that in the real printing process, including stress relaxation effects that are present at elevated temperatures. The material model has been calibrated to experimental stress–strain curves, varying the strain rate as well as the temperature. See the result of the calibration in Figure 7. Although the material model is calibrated using wrought annealed alloy 718 materials, the evolution in dislocation density would behave similarly and should present a good representation of the stress–strain state after the printing process.

### 3.2. Inherent Strain Model

In addition to the thermo-mechanical model, an inherent strain modeling approach is used to predict the resulting deformations of the bridges as well as stresses in the wall structure. The commercial software Simufact Additive 2022.1 is used for these simulations. Large deformations and an elastoplastic material behavior with a von Mises yield criterion are considered. The flow stress data used for this model is the alloy 718 powder data provided in the software and is similar to the flow stress data published in [32] for the as-printed alloy 718. The value of the virgin yield limit is considerably higher than what is used for the thermo-mechanical model [31]. The choice of a higher flow stress can be motivated by the fact that the ISM model does not take the actual process into account and therefore does not experience the same hardening due to the increase in plastic strain. A voxel mesh was used for all inherent strain simulations with a uniform size of 0.5 mm, where one layer of the mesh is equal to the modeled layer thickness.

The inherent strain model is based on the principle that a strain tensor, $\varepsilon_{ij}^{*}$, is applied to each added layer of the model. In the PBF-LB process, the inherent strain can originate from a variety of sources, such as thermally induced strain $\varepsilon_{ij}^{th}$, phase transformations $\varepsilon_{ij}^{ph}$, creep $\varepsilon_{ij}^{cr}$, and plastic strain $\varepsilon_{ij}^{p}$, i.e., the inherent strain tensor $\varepsilon_{ij}^{*}$ can be formulated as

(4) $$\varepsilon_{ij}^{*}=\varepsilon_{ij}^{th}+\varepsilon_{ij}^{ph}+\varepsilon_{ij}^{cr}+\varepsilon_{ij}^{p}$$

according to [5]. The total strain $\varepsilon_{ij}^{tot}$ can then be expressed as the elastic strain $\varepsilon_{ij}^{el}$ plus the inherent strain $\varepsilon_{ij}^{*}$

(5) $$\varepsilon_{ij}^{tot}=\varepsilon_{ij}^{el}+\varepsilon_{ij}^{*}$$

A quasi-static mechanical equilibrium is found to determine the stresses and distortions induced by the applied inherent strain in the building process

(6) $$\sigma_{ij,j}+b_{i}=0$$

where the $\sigma_{ij}$ is the stress tensor, and $b_{i}$ is the body forces per unit volume. The stress tensor can be computed according to

(7) $$\sigma_{ij}=D_{ijkl}\varepsilon_{kl}^{el}$$

where $D_{ijkl}$ is the elastic stiffness tensor.

**Figure 8:**
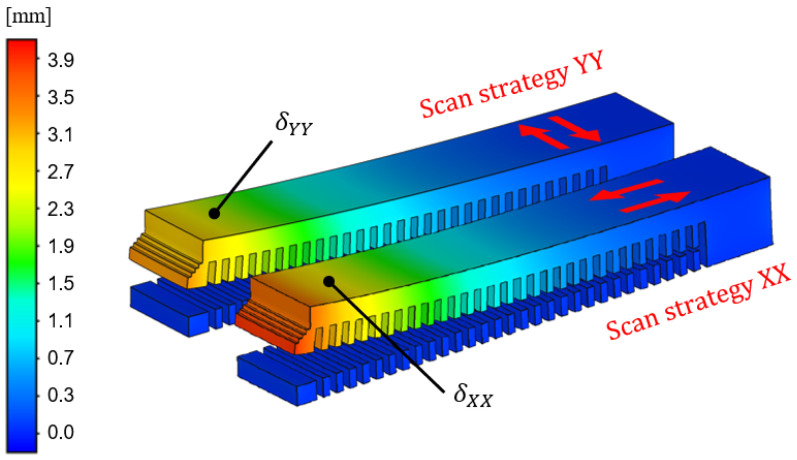
The z-deflection of the cantilevers after calibrating the inherent strain $\varepsilon_{ij}^{*}$ using the scanning strategy XX and YY, respectively. The red arrows indicate the scan strategy. The two points $\delta_{XX}$ and $\delta_{YY}$ show the position of the calibration points. The measurement was located 5 mm from the free end of the cantilever on the top surface.

The building process of the two types of cantilever beams with different scanning strategies (XX and YY) was simulated in an iterative procedure to find the inherent strain $\varepsilon_{ij}^{*}$. The calibration module in Simufact Additive was used to perform this iterative process. The module utilizes a Newton–Raphson-based optimization to minimize the error between the measured and modeled deflection until the relative error is less than 0.5 % for the calibration point on each of the two cantilever beams. The measured deflection was taken as an average of the CMM measurement points closest to the free end of the three printed cantilever beams for each scanning strategy. The average measured deflections were $\delta_{XX}=2.8\;\mathrm{mm}$ with a standard deviation of 0.13 mm and $\delta_{YY}=2.4\;\mathrm{mm}$ with a standard deviation of 0.073 mm for the longitudinal and transversal scanning, respectively. This resulted in the calibrated inherent strain values $\varepsilon_{xx}^{*}=-0.011$ in the longitudinal scan direction and $\varepsilon_{yy}^{*}=-0.007$ in the transverse scan direction. Figure 8 shows the simulated result for the deflection of the cantilever beams using the calibrated inherent strain. It is possible to simulate the XX and the YY scanning strategies by swapping the inherent strain values although a layer-by-layer approach is utilized.

The shear components of the inherent strain were set to 0. Thermo-mechanical models have previously shown that the shear components tend to be two or more orders of magnitude smaller than the normal components [6] and therefore only the more relevant normal components are typically used for part scale predictions of residual stresses using the ISM [4,6,33]. The zz-component of the inherent strain was not calibrated since it does not introduce any stresses to the underlying part because the upper nodes of each activated layer are unconstrained [33]. It has also previously been shown through sensitivity analysis that the zz-component does not influence the residual stresses [6].

## 4. Results and Discussion

**Figure 9:**
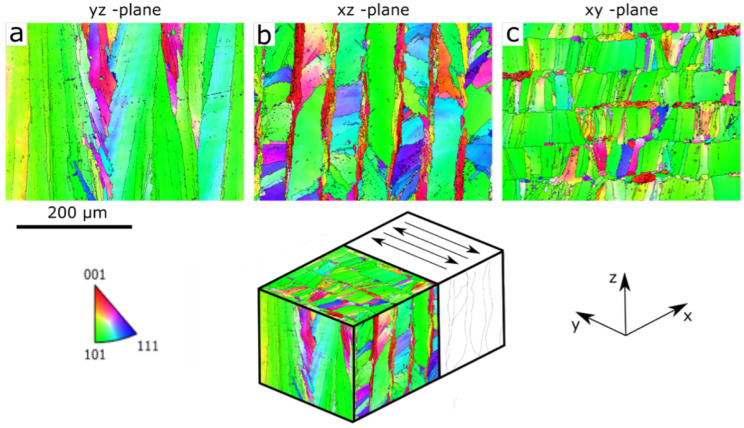
EBSD-IPF images showing the crystallographic texture in the different planes, (**a**,**b**) are planes (yz and xz) with a normal vector perpendicular to the build direction and (**c**) is a plane (xy) with its normal vector parallel to the build direction. All figures are colored as viewed along the z-axis.The arrows shown in the iso-metric view illustrates the scanning pattern.

In Figure 9, EBSD-IPF micrographs acquired from the yz, xz, and xy-plane, and colored as viewed along the z-axis, are presented together with a montage that illustrates the planes on an imaginary cube inside the wall. A pronounced [1 0 1] texture along the z-axis, i.e., the build direction, is seen for all planes. Figure 9b shows a distinct columnarity with alternating large and small grains at regular spacing. This type of grain structure has been reported earlier for FCC-alloys printed with similar scanning strategies [34] and is a result of smaller grains forming in the center of each melt pool. Phase analysis using the XRD data collected for residual stress measurements reveals that no phases except γ-Ni could be found in the material; see Figure 3.

### 4.1. Cantilever Deflections

**Figure 10:**
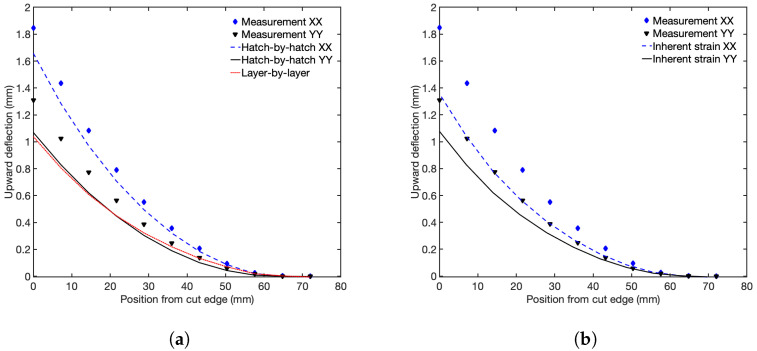
Measured and computed deflection of the bridges with scanning strategy XX and YY for (**a**) the thermo-mechanical models and (**b**) the inherent strain method. The measurement positions are from the left edge in Figure 2.

The modeled and measured upward deflections of the bridges are compared in Figure 10. Figure 10a shows modeling results from the hatch-by-hatch XX and YY scanning strategies. Additionally, it includes results from the layer-by-layer modeling strategy. Figure 10b presents results from the inherent strain model. The scanning strategy that is parallel to the length of the bridge (scanning strategy XX) results in a larger upward deflection compared with the scanning strategy that is perpendicular to the laser beam movement (scanning strategy YY). The experimental results agree with earlier observations, where the highest stresses are in the direction of the laser hatches [9,35].

The hatch-by-hatch and the ISM methodology successfully capture the variation in deflection depending on the used scanning strategy, although there is an overall underprediction. The maximum measured deflection is 1.84mm, applying the XX scanning strategy, which can be compared with the prediction of the hatch-by-hatch model of 1.65mm. This results in an error of 10%. For the YY scanning strategy, the measured and calculated deflections are 1.31mm and 1.06mm, respectively. This gives an error in the prediction of 19%. The predicted deflections for the ISM model are 1.35mm for the XX scanning strategy and 1.07mm for the YY scanning strategy, which is an underestimation of 27% and 18%, respectively. These results show an overall better prediction of the deflection when applying the thermo-mechanical hatch-by-hatch model than the ISM model. See Table 3 for a summary of the measured and calculated deflections and the predicted errors.

Although most of the parameters for the heat input model in Equation (2) are given by the process parameters, the efficiency factor η and the fictive heating time $t^{*}$ need to be calibrated. For alloy 625, it is found to be η=0.55 and $t^{*}=0.4\;s$, respectively, applying the layer-by-layer approach, a scanning speed of 1200mm/s, and a laser power of 100W [12]. Because alloy 718 has a very similar chemical composition compared with alloy 625, the absorption coefficient of the incoming laser is expected to be similar, and the determined values are used in this work as well. Furthermore, it is assumed that the efficiency factor is unaffected by the chosen lumping approach, which means that it is applicable to both the hatch-by-hatch approach and the layer-by-layer approach. It is also assumed that the efficiency factor and the fictive heating time are unaffected by the laser power and the scanning speed. However, these parameters may affect the true thickness of the powder layer, which indirectly may influence the model parameters, such as the efficiency factor and the fictive heating time, but this has not been taken into account.

It is known that the actual thickness of the deposited powder layer deviates from the theoretical thickness and as a result of shrinkage after melting and solidification, as well as spattering of the powder grains [36], it is generally higher. In the work by Mahmoodkhani [37], the effective powder thickness was measured to be in the range of 150 μm when printing a sample in stainless steel (SS17-4 PH powder from EOS GmbH) and a nominal build layer thickness of 20 μm with a scanning speed of 1108mm/s, a laser power of 227 W, and a hatch distance of 90 μm. This phenomenon has been confirmed in our lab to be in the same range for alloy 718 as well and may be one of the error sources in the deviation between modeled and experimental results.

### 4.2. Calculated and Measured Stresses in the Walls

**Figure 11:**
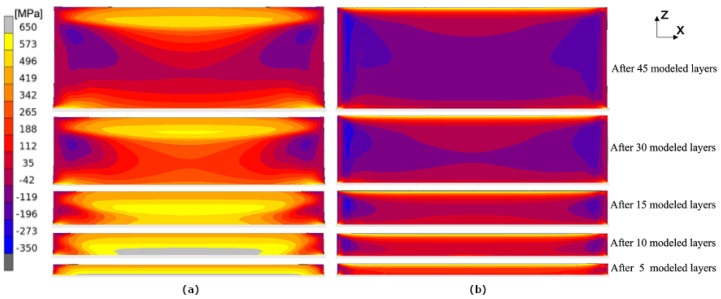
Evolution of $\sigma_{xx}$ during the building process. Results from the thermo-mechanical model using the layer-by-layer (**a**) and the hatch-by-hatch YY approach (**b**).

**Figure 12:**
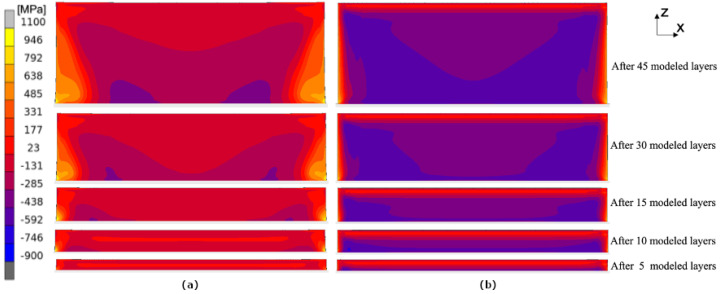
Evolution of $\sigma_{zz}$ during the building process. Results from the thermo-mechanical model using the layer-by-layer (**a**) and the hatch-by-hatch YY approach (**b**).

The calculated evolution of the normal stress $\sigma_{xx}$ during the building process applying the layer-by-layer approach and the hatch-by-hatch YY approach is presented in Figure 11. The calculated stress shown in the following figures is the mean stress over the thickness. This is performed in order to resemble the averaging of the stresses through the thickness due to the measurement method that is used. Figure 12 shows the evolution of the normal stress $\sigma_{zz}$ during the building process. The ’snapshots’ in both figures are taken at the end of the cool-down sequence, i.e., right before the scanning of the next layer starts. A similar trend can be seen when the two approaches are compared, i.e., the compressive and tensile stresses are confined to the same regions. For $\sigma_{xx}$, tensile stresses appear at the top and bottom with compressive stresses in the middle of the wall. For $\sigma_{zz}$, there are tensile stresses at the outer edges and compressive stresses in the middle of the wall.

In Figure 11, it is seen that the hatch-by-hatch approach results in a higher amount of compressive stresses than the layer-by-layer approach. The deviation between the layer-by-layer approach and the hatch-by-hatch approach is somewhat surprising since the two approaches agree well with each other in the bridge deflection results (see Figure 10). The difference may be a result of the cooling time between adding a new lumped segment. In the current version of the hatch-by-hatch and the layer-by-layer approach, the material is allowed to cool to near room temperature between each added segment. The temperature history impacts the relaxation mechanism that the material model accounts for, and therefore, it indirectly depends on the size and the geometry of the model.

**Figure 13:**
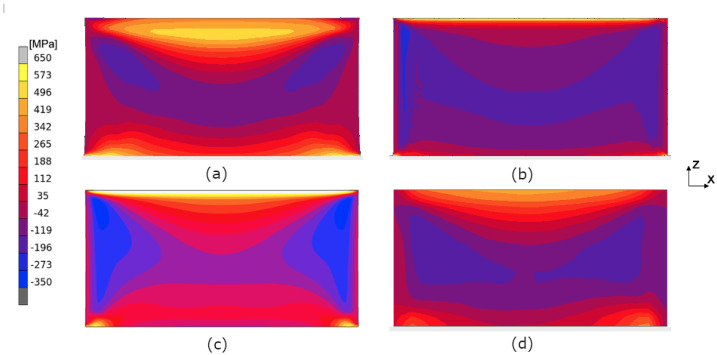
Residual stress $\sigma_{xx}$ in the finished wall (scanning strategy YY), modeled using (**a**) the thermo-mechanical layer-by-layer approach, (**b**) the thermo-mechanical hatch-by-hatch approach, and (**c**) the inherent strain method. The corresponding stress calculated from the synchrotron X-ray diffraction experiment is shown in (**d**).

Figure 13 presents the modeled and experimental residual stress fields for the normal stress $\sigma_{xx}$. The two thermo-mechanical modeling approaches end up in reasonably good agreement compared with the experimental results. However, a difference in the symmetry of the stress field can be seen; the hatch-by-hatch modeling approach shows a small asymmetry and is a result of the scanning strategy. An asymmetry in the stress field can also be seen in the experimental results. The ISM shows good agreement with the experimental result and with the thermo-mechanical results.

**Figure 14:**
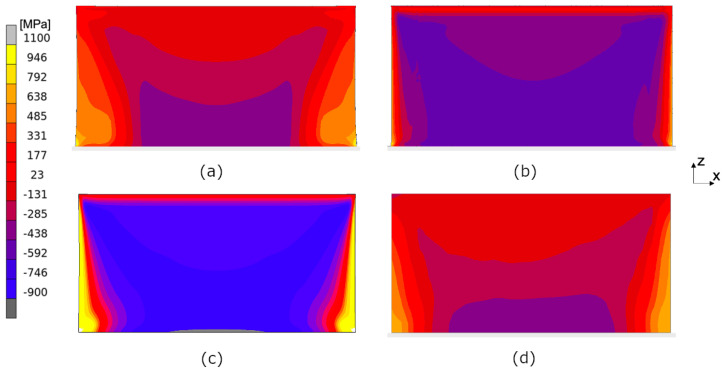
Residual stress $\sigma_{zz}$ in the finished wall (scanning strategy YY) modeled using (**a**) the thermo-mechanical layer-by-layer approach, (**b**) the thermo-mechanical hatch-by-hatch approach, and (**c**) the inherent strain method. The corresponding stress calculated from the synchrotron X-ray diffraction experiment is shown in (**d**).

Figure 14 shows modeled and experimental residual stress fields for the normal stress $\sigma_{zz}$. In this case, the modeled stress field by the layer-by-layer approach is closer to the experimental results. However, given the similarities between the layer-by-layer and the hatch-by-hatch approaches when predicting the deflection of the bridges, one may expect similar stress fields. The ISM shows poor agreement with the experimental result for this stress direction.

The experimental stress extraction procedure assumes a plane stress condition. However, the modeling results reveal a slight variation in the stress distribution across the thickness of the sample. It is important to keep this discrepancy in mind when comparing the results. The modeled stress fields, as illustrated in Figure 11, Figure 12, Figure 13 and Figure 14, are obtained from an xz-plane located at the center of the wall. In contrast, the gauge volume in the measurements extends throughout the entire thickness of the wall. This difference in location may lead to measured stress values that are lower than expected because the stress levels are highest in the center of the wall and decrease towards its surfaces.

We have shown here that the different solution strategies give various results, where the inherent strain methodology deviates most from experimental findings. However, this solution strategy is much faster than the thermo-mechanical solution strategies. For example, the computation time is approximately 10min for the wall (the calibration takes about 2h). The solution time for the layer-by-layer is about 24h and, for the hatch-by-hatch, it is in the order of 1week. Solving the bridge with modeled YY hatches takes almost 20days. All simulations were performed on Intel^®^Xeon^®^Gold 6248R 3.0 GHz CPU.

## 5. Conclusions

In this study, we focus on simulating residual stresses in alloy 718 samples manufactured through PBF-LB technology. We employ a thermo-mechanical model, which naturally considers relaxation effects integrated into the mechanism-based material model for describing the material’s flow stress behavior. Additionally, we utilize an inherent strain finite element model, calibrated using cantilevers printed with two distinct scanning strategies: parallel or perpendicular to the long side of the structure. This calibration allows us to account for the influence of the laser scanning direction.

To address the scanning direction in the thermo-mechanical model, we developed a hatch-by-hatch modeling strategy, which we compare with the more commonly used layer-by-layer approach. Furthermore, we compare our simulated results with experimental deformation measurements and high-energy X-ray diffraction residual stress measurements. The key findings from our research are as follows:

- The larger deflections measured for the bridges printed with a laser scanning direction parallel to the long side of the structure (scanning strategy XX), and the smaller deflection when the laser scanning direction was perpendicular to the long side of the structure (scanning strategy YY), are successfully predicted by the hatch-by-hatch and by the inherent strain modeling strategy. The layer-by-layer modeling approach is not capable of predicting different results in different scanning directions. This is a result of the isotropic thermal strains assumed in the thermo-mechanical layer-by-layer method, while the inherent strains method, which is also a layer-by-layer method, has different applied strains in the longitudinal and transversal directions.
- The computed deflections of the bridges are underestimated compared with the experimentally measured values. The results show an overall better prediction of the deflection applying the thermo-mechanical material model compared with the inherent strain model. It should be noted that the thermo-mechanical model is not calibrated/tuned at all in this work. The model parameters are all derived from a previous work [12], while the inherent strain model is calibrated for the current material and process parameters. A ’better’ result could for sure have been achieved with some tuning, but this shows the true predictability of the proposed modeling approach.
- All three modeling approaches capture the general appearance of the experimentally measured $\sigma_{xx}$ stress field in the wall, although the hatch-by-hatch modeling approach predicts the asymmetry that is experimentally detected as a result of the scanning direction. The layer-by-layer modeling approach predicted the $\sigma_{zz}$ stress field very well, whereas the two other strategies predicted that stress field with less accuracy.

The laser energy density is believed to influence the amount of spatter during the PBF-LB process. This may affect the energy efficiency factor in the model, which is a critical parameter in the modeling of the PBF-LB processes. In order not to introduce any tuning of the thermo-mechanical models in the current work, the process parameters from the work by [12] were kept the same in this work.

## Figures and Tables

**Table 1:** Chemical composition of the 718 alloys used in the PBF-LB process (wt%).

Ni	Cr	Fe	Mo	Nb	Ti	Al	Si	C	Mn
Bal.	18.7	18.0	3.0	5.14	0.94	0.42	0.05	0.028	0.07

**Table 2:** Number of printed and modeled layers and hatches. The layer thickness or hatch spacing is given in parentheses with mm unit.

	Physical	Physical	Physical	Modeled	Modeled	Modeled
	**Layers**	**Hatches XX**	**Hatches YY**	**Layers**	**Hatches XX**	**Hatches YY**
Bridge	625 (0.02)	83 (0.06)	1250 (0.06)	50 (0.25)	10 (0.5)	149 (0.5)
Wall	1500 (0.02)	66 (0.06)	1000 (0.06)	60 (0.5)	8 (0.5)	120 (0.5)

**Table 3:** Measured and calculated maximum deflection of the bridges given in mm. Error between the measured and calculated deflection is given in the parentheses.

Scanning Strategy	Experimental Results	Hatch-by-Hatch	Layer-by-Layer	Inherent Strain
XX	1.84	1.65 (10%)	1.05 (43%)	1.35 (27%)
YY	1.31	1.06 (19%)	1.05 (20%)	1.07 (18%)

## Data Availability

The data presented in this study are available on request from the corresponding author. The datasets presented in this article are not readily available due to technical limitations.
